# Greenland shark (*Somniosus microcephalus*) feeding behavior on static fishing gear, effect of SMART (Selective Magnetic and Repellent-Treated) hook deterrent technology, and factors influencing entanglement in bottom longlines

**DOI:** 10.7717/peerj.4751

**Published:** 2018-05-17

**Authors:** Scott M. Grant, Rennie Sullivan, Kevin J. Hedges

**Affiliations:** 1 Centre for Sustainable Aquatic Resources, Memorial University of Newfoundland, St. John’s, NL, Canada; 2 Central and Arctic Region, Arctic Aquatic Research Division, Fisheries and Oceans Canada, Winnipeg, MB, Canada

**Keywords:** Greenland shark, Longlines, Shark deterrent technology, Bycatch mitigation, Inertial suction, Feeding behavior, Feeding kinematics

## Abstract

The Greenland Shark (*Somniosus microcephalus*) is the most common bycatch in the Greenland halibut (*Reinhardtius hippoglossoides*) bottom longline fishery in Cumberland Sound, Canada. Historically, this inshore fishery has been prosecuted through the ice during winter but winter storms and unpredictable landfast ice conditions since the mid-1990s have led to interest in developing a summer fishery during the ice-free season. However, bycatch of Greenland shark was found to increase substantially with 570 sharks captured during an experimental Greenland halibut summer fishery (i.e., mean of 6.3 sharks per 1,000 hooks set) and mortality was reported to be about 50% due in part to fishers killing sharks that were severely entangled in longline gear. This study investigated whether the SMART (Selective Magnetic and Repellent-Treated) hook technology is a practical deterrent to Greenland shark predation and subsequent bycatch on bottom longlines. Greenland shark feeding behavior, feeding kinematics, and variables affecting entanglement/disentanglement and release are also described. The SMART hook failed to deter Greenland shark predation, i.e., all sharks were captured on SMART hooks, some with more than one SMART hook in their jaw. Moreover, recently captured Greenland sharks did not exhibit a behavioral response to SMART hooks. In situ observations of Greenland shark feeding show that this species uses a powerful inertial suction mode of feeding and was able to draw bait into the mouth from a distance of 25–35 cm. This method of feeding is suggested to negate the potential deterrent effects of electropositive metal and magnetic alloy substitutions to the SMART hook technology. The number of hooks entangled by a Greenland shark and time to disentangle and live-release a shark was found to increase with body length.

## Introduction

Cumberland Sound is a large (ca. 250 × 80 km) inlet located on the east coast of Baffin Island, in the Arctic territory of Nunavut, Canada. Since 1986, the inshore management area of Cumberland Sound has supported a small scale winter longline fishery for Greenland halibut (*Reinhardtius hippoglossoides*) (Fisheries and Oceans Canada (DFO), 2008). The fishery was initially licensed annually under experimental or exploratory licenses and has been treated as a commercial fishery since a quota of 500 t was established in 1994 (Fisheries and Oceans Canada (DFO), 2008). Local interest in this fishery from the indigenous community of Pangnirtung grew rapidly with peak participation (115 fishers) and landings (430 t) in the early 1990s (Fisheries and Oceans Canada (DFO), 2008). Historically, the fishery has been prosecuted during the winter (January–May) when land fast ice allows access to deep water (>400 m) which is the preferred habitat of Greenland halibut (Bowering & Nedreaas, 2000; Fisheries and Oceans Canada (DFO), 2008). Increasingly shorter sea-ice seasons, less stable ice conditions, and a winter storm in 1996 which resulted in a 70% loss of fishing gear all contributed to a substantial reduction in participation and landings in the 2000s with a low of six fishers and 3 t in 2007 (Dennard et al., 2010; Fisheries and Oceans Canada (DFO), 2008). Consequently, there is an increasing interest in developing a more stable and safer summer fishery during the ice-free season (July–October). Further, with the aim of developing economic and food security for Arctic Canada exploratory longline surveys to determine the commercial potential of Greenland halibut are proposed for the several fjords located on the east coast of Baffin Island.

The Greenland shark (*Somniosus microcephalus*) is the largest fish species in the Arctic Ocean and the only species of shark to occur in Arctic waters year-round (Compagno, 1984). The Greenland shark is the most common bycatch in the Cumberland Sound winter longline fishery for Greenland halibut (Fisheries and Oceans Canada (DFO), 2008; Young, 2010). All Greenland sharks are discarded since the toxicity of their flesh (MacNeil et al., 2012) precludes commercial sales. Fishers participate in a voluntary logbook program and from 1987 to 2006, reported catches of Greenland shark in the winter Greenland halibut fishery ranged from 0.4 to 2.9 sharks per 1,000 hooks (mean, 1.1/1,000 hooks) (Fisheries and Oceans Canada (DFO), 2008). The bycatch of Greenland shark was found to increase substantially (i.e., 6.3 sharks per 1,000 hooks) during an experimental longline fishery for Greenland halibut that took place in Cumberland Sound during the ice-free season in 2009 (Young, 2010). During this experimental fishery, a total of 570 Greenland sharks were captured incidentally. This bycatch of Greenland shark was estimated to be 4.8× the biomass of Greenland halibut landed (i.e., 35 t). Greenland sharks commonly entangle within longline gear and badly tangled sharks are often killed by fishers (Idrobo, 2008). About 50% of the sharks captured in the 2009 experimental summer fishery were released alive (Young, 2010) however post-release survival is unknown.

The International Union for the Conservation of Nature (IUCN) listed the Greenland shark as Near Threatened on the basis of possible population declines and limited knowledge of life history characteristics (IUCN, 2014). It has recently been suggested that Arctic populations of Greenland shark are not under conservation stress (MacNeil et al., 2012). However, much of our current understanding of the distribution and abundance of Greenland shark is limited to bycatch information in commercial fisheries and there is an inherent danger to drawing conclusions from commercial fishery data. Specifically, fisheries target aggregations of fish whose densities are determined by fish behavior not abundance (Rose, 2007). In addition, recent studies suggest late maturation (156 years) and extreme longevity (272 years) in the Greenland shark (Nielsen et al., 2016), life history characteristics that make them highly vulnerable to overfishing. Moreover, the general lack of knowledge on reproduction and factors influencing recruitment to spawning biomass of Greenland sharks supports erring on the side of caution by making every effort to avoid incidental harm. Sustainable resource use involves identifying ways to preserve the unique Arctic ecology and there is a need to manage Greenland shark bycatch (Food and Agriculture Organization of the United Nations (FAO), 1999; Davis et al., 2013).

In recent years, one of the most studied methods to mitigate the bycatch of sharks in longline fishing gear is the use of feeding deterrents that exploit the electrosensory system of sharks. Sharks possess a complex and extensive electrosensory system comprised of the ampullae of Lorenzini that are located around the snout or rostral area (Kajiura & Holland, 2002). This sensory system allows sharks to detect and localize weak bioelectric fields during the final stages of prey capture and they can also detect fish that are buried in sediments (Haine, Ridd & Rowe, 2001; Kalmijn, 1971; Kajiura, 2003; Kajiura & Holland, 2002). Demersal sharks that feed on or near the seabed and at depths where visibility is limited or under conditions of total darkness (i.e., >1,000 m) are more likely to rely on their olfactory, acoustico-lateralis, and electrosensory modalities. The Greenland shark is distributed to depths of 2,200 m (Herdendorf & Berra, 1995) and commonly exhibits a white snout caused from abrasion while foraging on the seabed suggesting it falls within this group. Moreover, their relatively small eyes (Bigelow & Schroeder, 1948) and parasite induced visual impairment possibly to the point of blindness in Arctic and subarctic populations (Berland, 1961; Borucinska, Whiteley & Benz, 1998) suggest they may rely heavily on their electrosensory system during the final stages of prey capture. Furthermore, Greenland halibut is a favored prey of Greenland shark (Yano, Stevens & Compagno, 2007), it buries within bottom sediments, and its depth distribution to 2,200 m (Templeman, 1973; Boje & Hareide, 1993) overlaps that of the Greenland shark.

Several studies have investigated the utility of electropositive metals (EPMs) and magnets to deter feeding, repel, and subsequently reduce the bycatch of sharks in longline fisheries (Brill et al., 2009; Godin et al., 2013; Hutchinson et al., 2012; Kaimmer & Stoner, 2008; O’Connell et al., 2010, 2011b, 2014; Rigg et al., 2009; Robbins, Peddemors & Kennelly, 2011; Stoner & Kaimmer, 2008; Tallack & Mandelman, 2009; Wang, McNaughton & Swimmer, 2008). There is evidence to suggest that when some species of sharks enter the electromagnetic field produced by EPMs and magnets they are repelled to some degree however results are mixed. It has been suggested EPMs and magnets are more likely to be effective where visibility is limited (Hutchinson et al., 2012) as in deep water habitats and for solitary sharks or sharks that occur at low densities and are less likely to interact vigorously (O’Connell et al., 2010; Jordan, Mandelman & Kajiura, 2011; Robbins, Peddemors & Kennelly, 2011). However, the primary mode of feeding (i.e., ram biting or suction) and ability/inability to adjust the prey capture sequence (Motta & Wilga, 2001) is also likely to be an important factor determining the effect of EPMs and magnets (Hutchinson et al., 2012). For example, studies suggest species that cannot readily adjust their feeding behavior during the final stages of the prey capture sequence are less likely to be repelled by the electromagnetic fields produced by EPMs and magnets (Hutchinson et al., 2012).

Challenges with regard to fishery applications of EPMs and magnets include the development of shark deterrent technologies that have a broad between species application and limit interfering with the operational and economic efficiency of commercial fisheries. By combining both an EPM and magnetic alloy on the same hook the SMART (Selective Magnetic and Repellent-Treated) hook has the potential to be broadly applicable to several shark species and eliminates complicated baiting configurations identified as an obstacle to commercial fishery applications (Robbins, Peddemors & Kennelly, 2011). In addition, the SMART hook technology has the potential to cope with species-specific deterrent effects of various EPMs and magnets by facilitating selective substitution once the most effective alloys have been identified. One potential limitation of this technology is the small size and subsequently small effective electromagnetic field.

This study investigated whether the SMART hook is a practical technology for reducing the capture of Greenland shark on bottom longlines that target Greenland halibut. Analysis included capture rates in SMART hook longline experiments, in situ behavioral bioassays on the effect of the SMART hook, and dissolution of the EPM component of the SMART hook. Greenland shark feeding behavior on static bottom fishing gear is also described for the first time and helps to provide a greater understanding of the limitations of longline feeding deterrents that exploit the electrosensory system. In addition, factors influencing entanglement in longlines and time required to disentangle and release Greenland sharks are also discussed.

## Materials and Methods

The current study was part of a multiyear (2011–2013) gear comparison study aimed at mitigating the capture of Greenland shark in Nunavut’s Greenland halibut longline fisheries. SMART hook longline experiments and SMART hook behavioral bioassays were conducted in Cumberland Sound during the ice-free season while onboard the *RV Nuliajuk*, a 19.8 m Nunavut research vessel that was crewed by experienced Greenland halibut longline fishermen. SMART hook longline experiments were carried-out in August 2011 and accompanied an annual longline research survey for Greenland halibut that commenced in Cumberland Sound in 2011. Variables affecting entanglement and release of Greenland sharks were obtained from the 2011 experimental and research survey longlines. In situ bioassays on the effect of the SMART hook on Greenland shark behavior were carried-out on jaw-hooked sharks that were captured on standard hooks during calm weather conditions. To obtain sufficient numbers of sharks, bioassays were conducted throughout the multiyear gear comparison study (i.e., 2011–2013). In 2012, an archived underwater video of Greenland shark feeding on bait suspended in a pot was brought to our attention. This video was from an exploratory fishery for porcupine crab (*Neolithodes grimaldii*) that was carried-out in subarctic waters in 1994 (He, Ennis & Walsh, 1994). This video was used in the current study to describe Greenland shark feeding behavior on static fishing gear.

### Longline experiment

**Figure 1:**
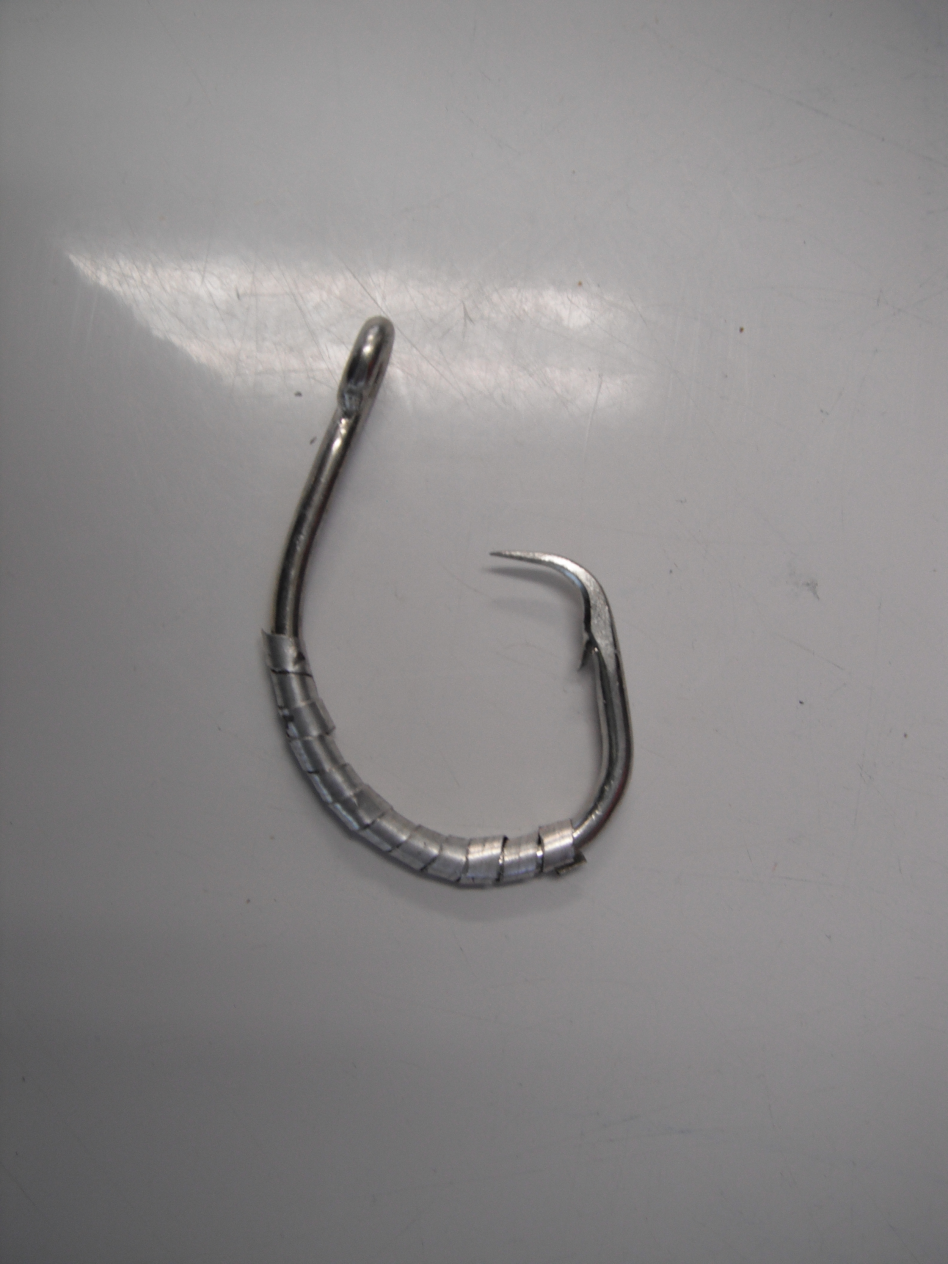
A circle 15/0 SMART hook (source: S. Grant).

Catches of Greenland shark were compared among Mustad circle 15/0 SMART hooks (20 mm gap size; Fig. 1) and standard Mustad circle 14/0 hooks (15.4 mm gap size). All hooks were made of carbon steel and had a 0° offset. Carbon steel circle hooks are used in open-water fisheries for Greenland halibut with hook size ranging from 14/0 to 16/0. The SMART hook was coated with Duratin(R) to resist corrosion in saltwater and specially magnetized to prevent entanglements with other fishing tackle. In addition, each SMART hook was wrapped with a 0.5–0.6 g strip of magnesium metal measuring approximately 250 × 3 × 0.3 mm (Fig. 1).

The experimental longline consisted of 200 hooks, 100 each of the standard and SMART hooks. Gangions were of braided nylon with a 118 kg breaking strength, 0.6 m in length, and attached to the mainline by Mustad rotor swivels at 1.8 m intervals. To ensure equal representation of hook types across the gear they were arranged in alternating groups of 20 (i.e., 20 SMART hooks, 20 standard hooks, etc.). All hooks were hand baited with frozen squid of similar size to that used in Greenland halibut longline fisheries. To reflect the depth distribution of Greenland shark and depth range of the winter and summer longline fisheries for Greenland halibut three experimental longlines were set at depths of 300, 500, and 960 m. As is typical in open-water commercial fisheries the experimental longlines were soaked overnight with soak time ranging from 14 to 16 h.

The number of hooks used in the experimental longline (i.e., 200) was similar to the number of hooks used in the Cumberland Sound winter fishery for Greenland halibut. However, longline stings with many more hooks (1,000–2,500) are commonly used in open-water fisheries. Gangion material, length, and interval on the experimental longlines was similar to that generally used in Canada’s commercial longline fisheries for Greenland halibut. However, rotor swivels which prevent the gangion from becoming twisted and allow the gangion to rotate around the mainline are not commonly used in Greenland halibut bottom longline fisheries. Rather, the gangion is simply tied to the mainline. When Greenland shark are captured on bottom longlines they typically roll resulting in the gangion and mainline wrapping around the body and caudal fin (Pike, 1994; Idrobo, 2008). Rotor swivels were used in the current study in an effort to reduce the level of entanglement of Greenland sharks.

Research survey longlines consisted of 200 standard Mustad circle 14/0 hooks. The bait type and size as well as the gangion material, length, interval, and method of attachment to the mainline (i.e., rotor swivels) were the same as the experimental longline. In 2011, a total of 22 research survey longlines were hauled from overnight sets (14–16 h) that covered a depth range of 300–1,002 m in Cumberland Sound.

A catch label was assigned to each hook upon haul back of both the experimental and research survey longlines (i.e., bait present/absent, species captured, hook loss, hook entangled by shark). However, only the capture of Greenland shark and number of hooks entangled by Greenland shark are considered here. Greenland shark mode of capture (i.e., by jaw hook and/or entanglement), number of hooks in the jaw, and time required to disentangle and release a shark were also recorded. Because of their large body size, none of the Greenland sharks were hauled onboard the vessel during disentanglement and all sharks were completely disentangled prior to release (i.e., there was no trailing gear embedded in or wrapped around the body or tail). It was not possible however to remove hooks that were embedded in the jaw. Greenland shark were assigned to three total body length size categories (<3 m, 3–4 m, and >4 m). Although poorly understood, these size categories approximate the size at maturity in males (3 m; MacNeil et al., 2012) and females (>4 m; Yano, Stevens & Compagno, 2007).

The dissolution and fragmentation of the magnesium metal strip of SMART hooks used in the longline experiment was monitored daily. Hooks that exhibited corrosion, cracking, and fragmentation were recorded.

### In situ behavioral bioassays

Tests of the ability of various EPMs and magnets to elicit a behavioral response include laboratory observations on immobilized sharks, typically juveniles or small bodied adults (Stoner & Kaimmer, 2008; O’Connell et al., 2011a). These tests are generally considered to provide a rapid method of determining which EPM and magnetic alloys are suitable for more extensive at-sea trials. During these tests sharks are inverted in the water which places them in what may be considered an un-natural orientation and behavioral state (Brooks et al., 2011) that is characterized by immobility and torpor. This state is called ‘tonic immobility’ (Watsky & Gruber, 1990). The standard methodology with EPMs and magnets is to align the test material in an anterior–lateral position to the head of an inverted shark, slowly move the material toward the ampullae of Lorenzini, and observe the shark’s behavior. Results of these behavioral bioassays have included no reaction, bending away from the material laterally, and thrashing and violent arousal from tonic immobility (Rice, 2008; Stoner & Kaimmer, 2008; O’Connell et al., 2011a). However, Brooks et al. (2011) concluded that tonic immobility was an inherently stressful experience in juvenile lemon sharks as it appeared to disrupt the short-term ventilation efficiency. Moreover, mixed results with regard to deterrent effects of EPMs and magnets in laboratory behavior experiments and lack of an impact on catch rates in longline experiments (Wang, McNaughton & Swimmer, 2008; Kaimmer & Stoner, 2008; Brill et al., 2009; Tallack & Mandelman, 2009; Robbins, Peddemors & Kennelly, 2011) suggest the possibility of a heightened response when sharks are caught off guard in tonic immobility. Thus, in some situations in situ analysis of behavior on recently captured sharks that are maintained in an upright orientation may better reflect the natural response to EPMs and magnets. Moreover, placing a shark in a state of tonic immobility may not be required when testing a species like the Greenland shark which has been reported to exhibit lethargic behavior under natural conditions (Watanabe et al., 2012) and no resistance when captured (Bigelow & Schroeder, 1948).

**Figure 2:**
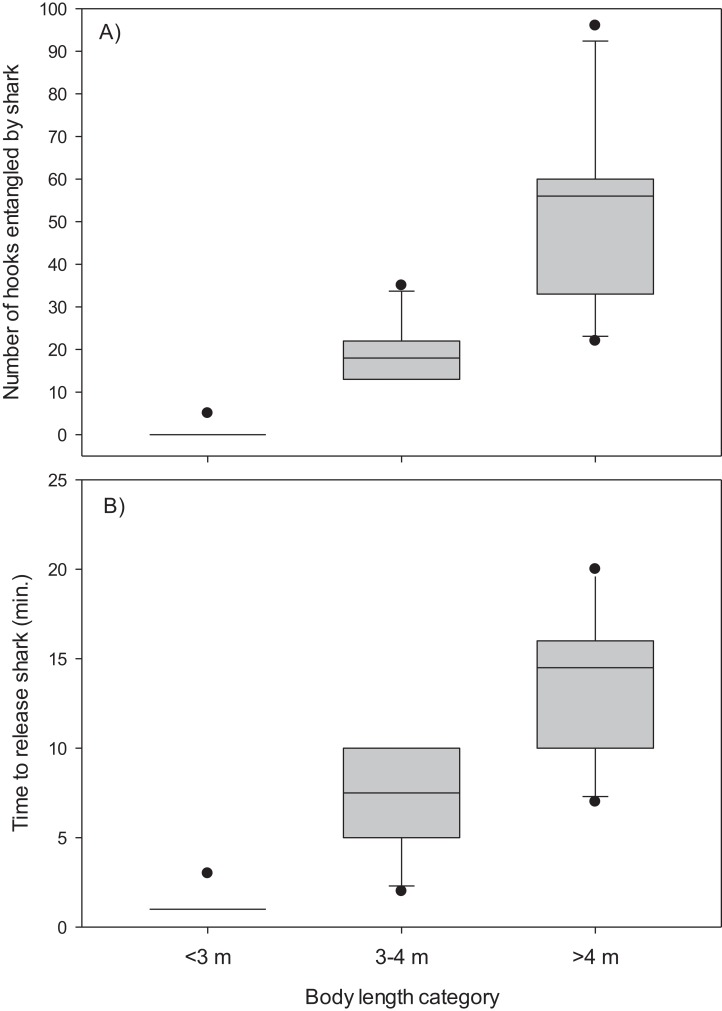
Box plots. (A) Number of hooks entangled by Greenland sharks and (B) time to release sharks for three body length categories (<3 m, *n* = 15; 3–4 m, *n* = 6; >4 m, *n* = 6).

In the current study, the behavioral response of 14 Greenland sharks that were captured by a single standard hook in the jaw and were not entangled in the longline were observed when they were exposed to (1) a SMART hook (Fig. 1) and (2) a 3.4 g clump of magnesium metal strips from six SMART hooks that were loosely wrapped around a stainless steel clip. The clump of magnesium strips was used to increase the voltage. During testing, sharks were exposed to the SMART hook followed by the clump of magnesium metal strips. During each trial the test material was lowered into the water on a wooden or fiberglass pole that was extended 0.75–1.25 m from the side of the vessel. Subsequently, the hook or clip was slowly moved laterally to within 2–5 cm of the snout of an upright shark from a distance of 0.50–0.75 m and at approximately 30° from the longitudinal axis of the body of the shark. The behavior of each shark was observed and the type of response recorded (i.e., no response, bend away, sudden movements of the caudal fin). All tests were completed within 1–2 min of the shark reaching the surface of the ocean.

The voltage of the SMART hook and clump of magnesium metal strips was measured using a Klein CL1000 digital multimeter (Klein Tools, Lincolnshire, IL, USA). Voltage measurements were obtained in seawater (34.6 ppt; 3.2 °C) by connecting one electrode to the SMART hook or clip of magnesium strips and the other electrode was attached to biological tissue (i.e., dorsal fin clip) from a Greenland shark. This methodology is similar to that used by O’Connell et al. (2014). A model IDR-309-T Gaussmeter with transverse probe (F.W. Bell, Milwaukie, OR, USA) was used to obtain the magnetic flux at two locations on the SMART hook (i.e., eye and point of the hook).

### Feeding behavior

Five underwater video sequences (4:18 min total) of a Greenland shark interacting with a baited pot were examined to determine the mode of feeding (i.e., ram bite vs suction) and feeding kinematics on static fishing gear. The shark was videotaped with a low speed (30 fields s^−1^) Xybion ISS 255 video camera designed to perform in harsh environments including low-light level underwater conditions (He, Ennis & Walsh, 1994). The camera was mounted 1.5 m above the center of a large (1.83 × 1.83 × 0.76 m; L × W × H) metal framed pot that was deployed on the slope of the Newfoundland-Labrador Shelf (Lat. 55°31.55′N, Long. 58°53.23′W) at a depth of 878 m. Illumination was provided by a 24 W incandescent light masked with a red filter to minimize the effect of light on animal behavior. The pot was baited with squid and herring that was suspended on skivers.

In the video footage, the movement of suspended particles by bottom currents was used to determine the approach direction (up or down current) of Greenland shark relative to the bait. Dimensions of the metal frame of the pot provided a means of obtaining estimates of the length of the shark and distance between the shark and the bait as the shark fed.

### Data analysis

Greenland shark capture data from the experimental and research survey longlines were combined for analysis of the effect of body size category on the number of hooks entangled by a shark and time to release the shark. Tests of normality and equality of variance were performed for each shark size category with the Kolmogorov–Smirnov normality test and the Levene median test, respectively. When assumptions of normality and equality of variance could not be met by transformation we used the non-parametric Kruskal–Wallis ANOVA on ranks. When this analysis indicated significant differences among size categories a Games–Howell multiple comparison procedure was used to test all pair wise comparisons. Statistical analyses were performed using SPSS® Statistics Version 19 (IBM, Armonk, NY, USA). Significance level was set to 0.05.

The project was reviewed and approved by the Freshwater Institute Animal Care Committee (Project # FWI-ACC-2011-045).

## Results

### Experimental and research survey longlines

A total of 27 Greenland sharks were captured in 2011 (Table 1). Six sharks were captured on three experimental longlines (600 hooks total) and 21 sharks were captured on 22 Greenland halibut research survey longlines (4,400 hooks total). Overall, sharks in the <3 m body length category dominated the catches accounting for 56% of the Greenland shark captured (Table 1). The SMART hook longline experiments were halted after three overnight sets owing in part to high numbers of SMART hooks entangled by Greenland sharks and subsequently loss or damage of hooks during disentanglement, dissolution and fragmentation of the magnesium metal strips of SMART hooks, and the capture of sharks with more than a single SMART hook in the jaw. In addition, results of the behavioral bioassays and observations of Greenland shark feeding behavior led to a decision to cancel additional SMART hook longline experiments in 2013.

**Table 1:** Greenland shark catch summary in experimental and survey longlines.

Longline type	Hook type	Number of hooks in jaw	Length category	Number of hooks entangled	Time required to release shark (min)
Experimental	SMART	1	<3 m	0	<1
	SMART	0^a^	3–4 m	15	10
	SMART	1	3–4 m	35	5
	SMART	2	>4 m	33	15
	SMART	2	>4 m	96	14
	SMART	2	>4 m	52	16
Survey	Standard	0^b^	<3 m	0	<1
	Standard	0^b^	<3 m	0	<1
	Standard	0^b^	<3 m	0	<1
	Standard	1	<3 m	0	<1
	Standard	1	<3 m	0	<1
	Standard	1	<3 m	0	<1
	Standard	1	<3 m	0	<1
	Standard	1	<3 m	0	<1
	Standard	1	<3 m	0	<1
	Standard	1	<3 m	0	<1
	Standard	1	<3 m	0	<1
	Standard	1	<3 m	0	<1
	Standard	2	<3 m	0	<1
	Standard	3	<3 m	5	3
	Standard	1	3–4 m	13	5
	Standard	1	3–4 m	13	10
	Standard	1	3–4 m	22	10
	Standard	3	3–4 m	21	2
	SMART	1^c^	>4 m	60	7
	Standard	2	>4 m	22	10
	Standard	2	>4 m	60	20

**Notes:**

a Entangled in SMART hook section of experimental longline.

b Captured by single hook partially embedded in skin of tail.

c Shark captured by entanglement in survey longline but SMART hook embedded in jaw.

All six of the Greenland sharks captured in the experimental longlines were captured on SMART hooks. Two of these sharks had a single SMART hook in the jaw and three sharks had two SMART hooks in the jaw (Table 1). Double and triple jaw-hooked sharks were also captured in the Greenland halibut research survey longlines (Table 1). The sixth shark captured in the experimental longline did not have a hook embedded in its jaw. This shark was entangled within 15 hooks of a SMART hook section of the experimental longline. In addition, a Greenland shark that was captured by entanglement within a research survey longline had a SMART hook embedded in its jaw with a severed gangion which is indicative of previous feeding upon a baited SMART hook from an experimental longline.

The mainline was wrapped around the body and/or tail region of 13 (48%) of the Greenland sharks captured in the combined experimental and research survey longlines. The number of hooks entangled by these sharks ranged from five to 96 (mean, 34.4 ± 7.2 S.E.) and it required 2–20 min (mean, 9.8 ± 1.5 S.E.) to disentangle and release these sharks (Table 1). Entanglement of 5–96 hooks corresponds to 9–173 m of mainline (mean, 61.9 ± 12.9 S.E.). During disentanglement, all hooks had to be cut from the mainline and in two cases the mainline also required cutting to facilitate removal of all fishing gear prior to release. Cutting of the mainline resulted in destruction of over 250 m of mainline. All 27 sharks were released alive and there was no evidence of external damage (i.e., hemorrhaging) owing in part to the Greenland sharks thick skin. Analysis indicated body length was a good predictor of the number of hooks entangled by Greenland sharks (χ^2^_(2)_ = 23.90, *p* < 0.001) (Fig. 2). Post-hoc analysis indicated the mean number of hooks entangled differed significantly between all body length categories (i.e., <3 m vs 3–4 m, *p* = 0.005; <3 m vs >4 m, *p* = 0.009; 3–4 m vs >4 m, *p* = 0.049). Mean time required to release a shark was also found to differ significantly among body size categories (χ^2^_(2)_ = 23.20, *p* < 0.001) (Fig. 2). In this analysis release times of <1 min were standardized to a value of 1. Post-hoc tests revealed time to release a shark differed significantly between all body length categories (<3 m vs 3–4 m, *p* = 0.020; <3 m vs >4 m, *p* = 0.003; 3–4 m vs >4 m, *p* = 0.045).

After a single overnight set the magnesium metal strips of all SMART hooks were corroded. After two overnight sets the magnesium strips were observed to be brittle and minor cracking under baiting pressure. Magnesium strips on hooks subjected to three overnight sets were easily broken resulting in fragments being lost under simulated baiting pressure.

### SMART hook behavioral bioassays

The SMART hook was both electropositive and magnetic, generating 1.2 V and a magnetic flux of 88 G. The clump of magnesium strips had a marginally higher voltage (1.4 V) then the SMART hook. None of the 14 Greenland sharks tested exhibited a detectable change in behavior when exposed to the SMART hook or clump of magnesium metal strips. These sharks were captured on longlines hauled from a depth range of 600–1,125 m (mean, 841 m) and all sharks swam away without delay when released. Nine of the sharks tested were <3 m in length, three were 3–4 m, and two were >4 m. The behavior of all Greenland sharks captured during this study could be characterized as lethargic and none of the sharks exhibited resistance whether they were hooked by the mouth alone or when entangled within the longline. However, entangled sharks were noticeably disoriented when released and descended well below the surface of the ocean before they were observed to swim (i.e., tail beat). The calm and non-aggressive behavior of Greenland shark is further illustrated by a lack of resistance by a total of 96 Greenland sharks captured during our multiyear gear comparison studies (i.e., 2011–2013). This includes nine sharks (i.e., five <3 m in length, three 3–4 m, and one >4 m) that were captured by a single hook that was only partially embedded in the skin of the upper or lower lobe of the caudal fin. These tail hooked sharks were captured on longlines that were hauled back from depths of 500 to 1,125 m. Three of these tail hooked sharks were captured in 2011 (Table 1).

### Feeding behavior

Archived underwater video recordings captured images of a single large Greenland shark (3–4 m in length) approaching and feeding on bait suspended in a pot. Four separate approaches were recorded and the shark always approached the pot slowly and from down current. Two separate feeding events were recorded with the shark oriented ventral-laterally to the camera. Feeding was characterized as inertial suction. During each suction feeding event the shark approached the pot slowly, rotated to align its mouth with the suspended bait, and then exhibited five to eight successive suction actions over a period of approximately 20–24 s.

The feeding kinematics were similar for each suction action. Specifically, as the lower jaw was depressed the labial cartilages and upper jaw were observed to protrude anteriorly to effectively form a somewhat round and laterally enclosed mouth which served to direct the suction anteriorly. Each of the successive suction actions was accompanied by minor cranial elevation however the timing relative to lower jaw elevation/upper jaw protrusion was not discernable. Bulging of the pharyngeal cavity was also observed during each suction action. During a suction feeding event the mouth opening became larger and swelling of the pharyngeal cavity increased which appeared to effectively increase the suction force. Even though the shark was outside of the pot it was able to repeatedly draw the suspended bait through the mesh and into its mouth from a distance of about 25–35 cm. In addition, in one instance the shark was able to suck into its mouth a scavenging hagfish as it swam into the path of the suction force.

It is notable that the feeding event observed in the current study was impeded by the meshes in the pot. The observed shark would have ingested the prey much more quickly under natural conditions. The observed feeding time is therefore longer than the period in which a foraging shark would be exposed to the effects of a SMART hook.

## Discussion

In the current study, the SMART hook did not deter Greenland shark from feeding on bottom longlines. Few Greenland sharks were captured in the experimental longline yet all were captured on SMART hooks and three sharks preyed upon more than one SMART hook. In addition, a Greenland shark captured on a survey longline was found to have a SMART hook embedded in its jaw. Additional experimental fishing trials with SMART hooks were abandoned during our multiyear gear comparison study because of unfavorable results. Specifically, capture of Greenland shark only on SMART hooks, repeat feeding on SMART hooks, absence of a behavioral response to the SMART hook and clump of magnesium metal, fragmentation of the magnesium metal strips, and powerful inertial suction which allowed a Greenland shark to suck bait into its mouth from a distance of 25–35 cm. Not only do our results provide evidence that Greenland sharks are not affected by the SMART hook but also that their powerful and successive suction actions during a feeding event are likely to negate the deterrent effects of the SMART hook technology when initiated beyond the range of the electromagnetic field produced by EPMs and magnets.

Studies show that some shark species exhibit aversion responses to EPM and magnetic alloys at distances of up to 100 cm, others do not respond until they are within 2 cm, while some species or individuals within an effected species show no response at all (Stroud, 2008; Stoner & Kaimmer, 2008; Brill et al., 2009; O’Connell et al., 2010; Robbins, Peddemors & Kennelly, 2011). As summarized by O’Connell et al. (2010), not all magnets and EPMs may be equally effective as repellents and not all shark species or individuals within a species may respond similarly to a specific alloy. Reasons for variability in repellent effects are unclear but may be related to several factors including the size, shape, and type of EPM or magnetic alloy used and subsequent electromagnetic field strength or how the fields are perceived by individual sharks (Brill, 2008; Rigg et al., 2009; O’Connell et al., 2010).

Large (215 × 100 × 67 mm) barium-ferrite magnets with a high magnetic flux (∼950 G) were found to alter the in situ feeding and swimming behavior of suction feeding nurse sharks (*Ginglymostoma cirratum*) when bait was placed within 30–50 cm of the magnets (O’Connell et al., 2010). Similarly, captive juvenile sandbar sharks (*Carcharhinus plumbeus*) avoided approaching closer than 100 cm to three large (100 × 20 × 20 mm) ingots comprised of neodymium rare-earth magnets and highly electropositive praseodymium (Brill et al., 2009). However, these large EPM and magnetic ingots would be unsuitable for use in longline fishing gear. More manageable sized barium-ferrite (25 × 25 mm) and neodymium–iron–boride magnets (25 mm × 12 mm) with a high magnetic flux (∼3,850 G) have been adapted for use in commercial longline and recreational hook-and-line fisheries (O’Connell et al., 2011b). However varying species-specific and within species deterrent effects were observed and the complicated baiting configuration would not be suitable for use in the Greenland halibut bottom longline fishery.

The low magnetic flux of the SMART hook used in this study produces a relatively weak magnetic field. Magnesium is a relatively weak EPM however, the electric voltage produced by the SMART hook was well above the nanovolt (10^−9^) threshold of sensitivity exhibited by sharks. Nevertheless, the SMART hook did not deter Greenland sharks from feeding on bottom longlines. Further, none of the Greenland sharks tested during our behavioral bioassays exhibited aversion behavior to the SMART hook even when the voltage was increased marginally through the use of a clump of magnesium metal strips.

Stress and physical exhaustion may influence the existence and magnitude of a behavioral response to EPMs and magnets. It is unclear what affect the stress of being held in captivity, netted, handled, and physically inverted to induce a state of tonic immobility that appears to interfere with respiration has on behavioral bioassays of sharks. In the current study, Greenland sharks were captured on longlines set at depths of 600–1,125 m. Greenland shark lactate levels were recently reported to increase with depth of capture on longlines (Barkley et al., 2017), but the lactate levels were highly variable and baseline reference levels are unknown for this species. Moreover, many of the sharks examined by Barkley et al. (2017) were entangled in the longline gear but the number of individual sharks entangled, number of hooks entangled around the body and tail, or time required to disentangle individual sharks prior to securing blood samples was not recorded (N. Hussey, 2018, personal communication). Thus, it is unclear whether the elevated lactate levels were the result of depth of capture or level of entanglement and time required to release sharks from longline gear. We recommend future physiological and tagging studies involving the capture of Greenland shark on longlines record and document whether sharks were entangled in the fishing gear, number of hooks entangled, and period of time required to disentangle sharks.

All of the sharks exposed to our behavioral bioassay were hooked by the jaw alone and did not appear stressed or physically exhausted as they were observed to immediately swim away from the vessel when released. Conversely, entangled sharks were noticeably disoriented when released and observed to descend several meters below the surface of the ocean before swimming. Because Greenland sharks tend to roll and entangle in longline gear (Pike, 1994; Young, 2010; current study) time of capture of sharks that are hooked by the jaw alone is likely to be shortly before haul back. Moreover, it is conceivable that many of the sharks that were hooked by the jaw alone were captured in the water column during haul back. For example, pelagic excursions of Greenland sharks are well documented (Skomal & Benz, 2004; Stokesbury et al., 2005; Campana, Fisk & Klimley, 2015), they have been captured at the surface of the ocean (Beck & Mansfield, 1969; Kondyurin & Myagkov, 1983), and during our multiyear gear comparison study we observed Greenland sharks at the surface of the ocean preying on Greenland halibut captured on longlines. The Greenland shark belongs to the family Somniosidae commonly referred to as sleeper sharks and the slow swimming, low activity level, and non-aggressive behavior of Greenland sharks is well documented (Bigelow & Schroeder, 1948; Watanabe et al., 2012). Further, free swimming Greenland sharks in the St. Lawrence Estuary have been described as docile during over 100 close encounters with divers and their tolerance to physical contact with sport divers including being captured by hook and line and lassoed by the tail has led to the development of a diver code of conduct (Greenland Shark and Elasmobranch Education and Research Group (GEERG), 2009). During the current study, lack of resistance or an escape response when hooked by the jaw alone or by a single hook only partially embedded in the skin of the tail and ability to survive when severely entangled in longline gear suggests a high threshold of tolerance and ability to cope with adverse conditions. Lastly, the calm behavior and immediate swimming response upon release exhibited by all jaw-hooked Greenland sharks captured on longlines during our gear comparison studies leads us to suspect that stress and exhaustion had little effect during our behavioral bioassays.

A reduction in the catch rates of spiny dogfish (*Squalus acanthius*) on SMART hooks in longline experiments carried-out in the Gulf of Maine provides evidence of the ability of this technology to deter feeding on baited hooks (O’Connell et al., 2014). Lack of evidence of a similar effect in the Greenland shark may be attributed to its powerful inertial suction mode of feeding when initiated beyond the range of the electromagnetic field produced by the SMART hook. It is unclear however whether this would account for all capture events on SMART hooks as suction feeding may not always be initiated from a suitable distance to avoid the electrosensory system from entering the electromagnetic field. A high threshold of tolerance to the effects of the SMART hook may account for the capture of Greenland sharks when the electrosensory system enters the electromagnetic field. However, effects of EPMs and magnetic alloys on the electrosensory system of sharks and rays are unclear and differing reactions among spiny dogfish and Greenland shark may be attributed to how the two species perceive the electromagnetic field. For example, the trophic level occupied by a shark and the diversity of predatory species in its local environment may be expected to influence the perception and response to an unfamiliar stimulus. In the Gulf of Maine, a region with a high diversity of species, the relatively small bodied spiny dogfish is considered to occupy a trophic level of 4.0 but has a high diversity of potential predators from birth (23–29 cm) to maximum length (100–120 cm) (Jensen, 1966; Nammack, Musick & Colvocoresses, 1985; Byron & Morgon, 2016). Thus, spiny dogfish may be more cautious and quickly repelled when encountering an unfamiliar electromagnetic field and subsequently unlikely to approach the same hook again. The Greenland shark is a reported 40–100 cm at birth, grows to a length of over 600 cm, and is the largest fish species in the Arctic Ocean (Compagno, 1984; MacNeil et al., 2012). The Greenland shark is a top predator occupying a trophic level of 4.2–5.0 in the Arctic (MacNeil et al., 2012), a region of comparatively low species diversity, and apart from accounts of cannibalism when captured on longlines (Borucinska, Whiteley & Benz, 1998) the Greenland shark has no known predators in Canadian Arctic waters. During our longline experiments Greenland sharks were only captured in the SMART hook section of the longline and the capture of sharks with a SMART hook already embedded in the jaw indicates repeat feeding on SMART hooks. These results and lack of a behavioral response to the SMART hook lead us to suggest not only a high threshold of tolerance to the unfamiliar stimulus caused by the SMART hook but also the possibility that Greenland sharks were positively stimulated by the weak electromagnetic field produced by the EPM and magnetic coating on the SMART hook used in the current study.

One of the features of the SMART hook is its ability to deal with species-specific deterrent effects of EPMs and magnetic coatings through selective substitution of these alloys. However, when the feeding behavior observed by Greenland shark in the current study and the apparent effective range of the electromagnetic fields of suitable sized EPM and magnetic alloys on other shark species is taken into consideration they raise concerns with regard to the utility of SMART hook substitutions. The magnetized SMART hook used in the current study possessed light weight (0.5–0.6 g) and relatively weak electropositive magnesium metal strips. When the aversion response in behavioral bioassays was assessed for comparatively larger ingots (70–100 g) of several types of highly EPMs and larger (102 × 38 mm) high magnetic flux rare-earth magnets it was found that the reactive distance of immobilized juvenile nurse sharks, lemon sharks (*Negaprion brevirostris*), and spiny dogfish ranged from 2 to 25 cm (Rice, 2008; Stoner & Kaimmer, 2008; Stroud, 2008). Similarly, free swimming captive spiny dogfish and juvenile dusky smooth-hound sharks (*Mustelus canis*) did not exhibit a negative response to the magnetic field produced by 25 mm square neodymium rare-earth magnets until they approached to within 10 cm. Overall, short reactive distances are not surprising because the detection range of the ampullae of Lorenzini is effective only within a few centimeters of electromagnetic fields as sharks utilize this sensory system in the final stages of capture to detect weak bioelectric fields generated by their prey.

To our knowledge the video of Greenland shark reported here represents the only documented underwater observations of Greenland shark feeding behavior and are relevant as the shark was scavenging bait from static fishing gear at the same depth longline fisheries prosecute Greenland halibut. The Greenland shark exhibited inertial suction and once a suction event was initiated it continued to completion. The Greenland shark was observed to exhibit several successive suction actions during a feeding event. This strategy is likely to increase feeding success especially when initiated from a distance in visually impaired Greenland sharks (i.e., ocular parasites) or when the prey attempts to escape. The increase in gape size and increased bulging of the pharyngeal cavity observed in this study would increase inertial suction forces during a feeding event and are likely to increase feeding success. Stealthy cryptic approaches and powerful suction would also explain how such a slow swimming shark (Watanabe et al., 2012) is able to consume Greenland halibut and small seals, especially when these animals are consumed whole and with no external damage. For example, stomach content analysis of a large (>4 m) Greenland shark captured on longlines set through the ice in Pond Inlet, Nunavut revealed the presence of a fully intact (i.e., no external wounds) and recently consumed 60 cm Greenland halibut and a fully intact 50–60 cm ringed seal (*Pusa hispida*) (R. Sullivan, 2006, personal observation). These relatively large animals appear to have been sucked directly into the large pharyngeal cavity and subsequently swallowed whole.

The Greenland shark is the largest member of the order Squaliformes or dogfish sharks which are morphologically specialized for suction feeding (Motta & Wilga, 2001). Greenland sharks commonly exhibit a white snout resulting from foraging on the seabed and sharks feeding on organisms that live on or within the seabed commonly utilize a suction mode of feeding (Motta & Wilga, 2001). Unlike ram and bite feeding sharks, suction feeders appear to have more stereotyped capture events with less ability to modulate between suction and ram type feeding during the prey capture sequence. Motta & Wilga (2001) proposed that suction captures will be preprogrammed stereotyped bites that go to completion once initiated, regardless of the sensory input. Fast swimming ram and bite feeding sharks appear to commit to attacking their prey from a distance and it has been hypothesized that when they execute the feeding sequence beyond the effective range of the electric field produced by EPMs the deterrent effects will be negated (Hutchinson et al., 2012). Similarly, we propose that the powerful inertial suction mode of feeding exhibited by Greenland shark will negate the deterrent effects of SMART hook EPM and magnetic alloy substitutions suitable for use in Greenland halibut fisheries. The reactive distance by two squalid shark species (nurse and spiny dogfish) to electromagnetic fields produced by several types of highly EPMs and high magnetic flux rare-earth magnets (Rice, 2008; Stoner & Kaimmer, 2008; Stroud, 2008) are within the suction range exhibited by Greenland shark in this study. When feeding on longlines, we suspect Greenland sharks use their olfactory and acoustico-lateralis systems to detect and orient to a bait plume and once in proximity are able to use their powerful and successive inertial suction forces to pull a baited hook off the seabed at a distance beyond that of the potential deterrent effects of current SMART hook technologies.

The feeding behavior of most shark species is poorly studied. This study illustrates that when the primary mode of feeding is taken into consideration it can provide a better understanding of potential limitations of longline feeding deterrents that exploit the electrosensory system. More recently, alternate longline modifications designed to reduce the incidental capture of Greenland shark have been tested with positive results (Munden, 2014). Development of methods that expedite and maximize live-release of entangled Greenland sharks from longlines and studies on post-release mortality will also be important to future management considerations. For example, in the current study the mainline, gangions, and hooks were completely disentangled from Greenland sharks prior to release. When entangled in commercial longlines, Greenland sharks are often released with trailing gear that is wrapped around the body or tail (S. Grant, 2014, personal observation). A recent study has demonstrated that when trailing fishing gear remains embedded in the tail of common thresher sharks (*Alopias vulpinus*) it can lead to high post-release mortality (Sepulveda et al., 2015).

The current study illustrates the degree of gear entanglement commonly caused by Greenland sharks when feeding on bottom longlines. Hooks that become entangle around the tail and body of the shark are unlikely to continue to lure and capture Greenland halibut (Dennard et al., 2010) and considerable time and gear will be lost disentangling Greenland sharks, particularly when bycatch rates are high. There is no way to determine when Greenland shark were captured during an overnight set and soak time may influence Greenland shark catch rates (Pike, 1994) and the degree of entanglement when sharks are captured near the seabed. Greenland shark are known to move throughout the water column (Skomal & Benz, 2004; Stokesbury et al., 2005; Campana, Fisk & Klimley, 2015), they have been taken at the surface by harpoon and in gillnets (Beck & Mansfield, 1969), and we have observed Greenland shark foraging at the surface and preying on Greenland halibut captured on longlines. These observations lead us to suspect that many of the Greenland sharks that are captured by a single hook in the jaw or tailfin are taken in the water column during haul back of the fishing gear. Sharks entangled in the mainline were clearly captured on the seabed as tension in the mainline during haul back and use of rotor swivels would preclude entanglement when Greenland sharks are captured within the water column. Cyclical vertical movements within the water column by the related Pacific sleeper shark (*Somniosus pacificus*) has been hypothesized to be a foraging strategy (Hulbert, Sigler & Lunsford, 2006) and adult Greenland halibut, a favored prey of Greenland shark, have been found to make regular excursions several hundred meters into the water column (Vollen & Albert, 2008). As a foraging strategy, vertical movements throughout the water column would help explain our observations of Greenland sharks feeding near the surface and high incidence of single jaw or tail hook modes of capture. If our hypothesis with regard to the pelagic capture of single jaw and tail hooked Greenland sharks is correct then it would appear that smaller Greenland shark were more likely to exhibit a pelagic distribution within Cumberland Sound during the ice-free season in 2011 as all of the non-entangled sharks were <3 m in length (Table 1). Kondyurin & Myagkov (1983) also reported a pelagic distribution for juvenile Greenland sharks that were <3 m in length. If larger and sexually mature sharks are closer to the seabed during the ice-free season then there is a greater likelihood they will become entangled in the fishing gear and be at greater risk of mortality.

## Conclusion

We conclude that the SMART hook is not a suitable technology for mitigating the capture of Greenland sharks on Greenland halibut bottom longlines. The SMART hook technology did not deter Greenland sharks from feeding on bottom longlines and this technology did not elicit a behavioral response in recently captured Greenland sharks. The Greenland shark was found to exhibit a powerful inertial suction mode of feeding and was able to draw food items into its mouth from a distance of at least 25–35 cm. Stealthy cryptic approaches and a powerful suction mode of feeding can explain Greenland sharks consumption of seals and fish. When initiated from beyond the effective range of the electromagnetic field this powerful suction is surmised to negate the effect of EPM and magnetic alloy substitutions to the SMART hook technology. Fragmentation of the magnesium metal strips and subsequently frequent replacement of SMART hooks is also identified as a limiting factor to commercial applications.

During the current study, interactions of Greenland sharks with bottom longlines led to entanglement of close to 50% of captures and at times entanglement was substantial. Even severely entangled Greenland sharks were alive when hauled to the surface from depths of up to 1,125 m and their lethargic behavior facilitated live-release efforts (i.e., removal of fishing gear). Commercial longline fishers commonly release Greenland sharks with trailing gear. Post-release survival of these sharks is unknown but expected to be low based on the results of related studies (Sepulveda et al., 2015). Until factors influencing post-release survival of Greenland sharks are better understood we recommend efforts be made to remove all trailing longline gear from Greenland sharks prior to release. During the current study, we avoided cutting the mainline while disentangling Greenland sharks which undoubtedly influenced the time required to release individual sharks. Hence, the mean time required to disentangle and release sharks reported herein is an overestimate of that expected under commercial conditions. Nevertheless, removal of trailing fishing gear will be a frustrating and time consuming process when bycatch rates of Greenland shark are high and sharks entangle large numbers of hooks. Further, economic costs associated with damage to and loss of fishing gear exemplifies the need to continue to investigate modifications to fishing gear, potential gear substitutions, or spatial management of fishing effort to reduce the incidental capture of Greenland sharks.

## Supplemental Information

10.7717/peerj.4751/supp-1 Supplemental Information 1 Greenland shark catch data.

10.7717/peerj.4751/supp-2 Supplemental Information 2 Greenland shark behavioural bioassay data.

## Additional Information and Declarations

### Data Availability

The following information was supplied regarding data availability: The raw data are provided in the Supplemental Files.
